# Screen Time Soars and Vision Suffers: How School Closures During the Pandemic Affected Children and Adolescents’ Eyesight

**DOI:** 10.1007/s10935-024-00800-3

**Published:** 2024-08-08

**Authors:** Muna Abed Alah, Sami Abdeen, Iheb Bougmiza, Nagah Selim

**Affiliations:** 1 Clinical effectiveness department, Primary Health Care Corporation, Doha, Qatar; 2 Community Medicine Department, Hamad Medical Corporation (HMC), Doha, Qatar; 3 Community Medicine Department, Primary Health Care Corporation (PHCC), Doha, Qatar; 4 Community Medicine Department, College of Medicine, Sousse University, Sousse, Tunisia; 5 Public health and Preventive Medicine Department, Cairo University, Cairo, Egypt

**Keywords:** Screen time, School closures, Students, Vision, Visual acuity

## Abstract

This study aimed to determine the impact of school closures on visual acuity and screen time among students in Qatar. An analytical cross-sectional study was conducted, targeting governmental school students. Data were collected via telephone interviews with parents, and visual acuity measurements were extracted from the electronic health records. We interviewed 1546 parents of selected students, about 24% reported their children’s history of visual disturbances, primarily refractive errors. The mean screen time across the week increased significantly by 11.5 ± 11.6 h during school closures. We observed a significant decline of visual acuity during the closure compared to the pre-closure period across the entire sample, both sexes, and the younger age group. Furthermore, logistic regression analysis showed that local students and those with a history of visual disturbances were 1.7 times (AOR: 1.73, 95%CI 1.18–2.54, *p* = 0.005) and 2.5 times (AOR: 2.52, 95%CI 1.69–3.76, *p* < 0.001) more likely to experience decline of visual acuity respectively. School closures in Qatar were associated with a significant increase in screen time among students and a notable decline in their visual acuity. This deterioration highlights the need to monitor children’s screen time and implement cost-effective measures to reduce screen exposure and enhance overall eye health among students.

## Introduction

The coronavirus disease 2019 (COVID-19) pandemic has had far-reaching effects on all aspects of daily life, including education. A significant consequence of this global crisis has been the widespread closure of schools and educational institutions worldwide(Abed Alah et al., 2020, 2021). While these measures were necessary to curb the spread of the SARS-CoV-2 (Severe Acute Respiratory Syndrome Coronavirus 2), they have profoundly impacted the vision and visual acuity of children and adolescents (Cortés-Albornoz et al., 2022). Extended periods of screen time reduced outdoor activities, and the stress related to the pandemic have collectively contributed to the deterioration of vision among this population. Consequently, growing concern revolves around the potential long-term effects of school closures on the vision and visual acuity of children and adolescents, thereby emphasizing the urgent need for interventions to mitigate further harm. Numerous studies have explored the alterations in screen time patterns during the COVID-19 pandemic and have consistently reported a substantial increase in screen time among children and adolescents, particularly during periods of home confinement and school closures when compared to pre-pandemic levels. According to a meta-analysis of 89 studies, there was a pronounced upsurge in both total and leisure screen time among primary-aged children and adolescents (Trott et al., 2022). Specifically, the study reported an average daily increment of 1.4 h and 0.9 h in total screen time for primary-aged children and adolescents, respectively. Moreover, leisure screen time (referring to non-academic screen based activities) showed an average daily increment of 1.0 h and 0.5 h for primary-aged children and adolescents, respectively (Trott et al., 2022). Mohan et al. (Mohan et al., 2021) reported an increase in the prevalence of Digital Eye Strain (DES) due to the surge in digital device usage and screen time during the COVID-19 pandemic. Their study indicated that over 50% of children who were participating in online classes experienced DES. In India, attending online classes resulted in a 51% prevalence of DES among students aged 7 to 16 years (Amarnath & March De Ribot, 2021). Additionally, in Saudi Arabia, a significant surge in device usage was observed during the COVID-19-related lockdown, with approximately70% of children exhibiting symptoms indicative of DES (Aldukhayel et al., 2022). Further evidence originates from routine checkups for students in China, revealing a decline in visual acuity during the COVID-19 pandemic, particularly in children who had pre-existing vision problems (Noi et al., 2022).

In this study, we assessed the impact of school closures during COVID-19 on the screen time and visual acuity of children and adolescents in Qatar. It is part of a larger national project that assessed the impact of COVID-19-related school closures on the lifestyle and vision of children and adolescents.

## Methods

### Study Participants

In this analytical cross-sectional study, we targeted children and adolescents enrolled in Qatar’s governmental schools, spanning from 3rd to 9th grades. Visual acuity screenings are routinely performed by school nurses, using Snellen charts with the results recorded in the electronic health records system. Data collection for this study occurred from June to August 2022. To ensure uniformity, we included only those students who underwent visual acuity tests after the schools’ reopening and compared these results with their previous visual acuity records taken before the closure, if available. The study has been ethically approved by the institutional review board (IRB) of Hamad Medical Corporation (MRC-03-21-895) .

### Study Procedure and Sampling Technique

To conduct this study, we obtained a comprehensive list of all students attending government schools in Qatar, encompassing grades 3 to 9. Based on sex and age, we stratified the students into four distinct strata: (1) males aged 8 to 11 years, (2) females aged 8 to 11 years, (3) males aged 12 to 15 years, and (4) females aged 12 to 15 years. This stratification was facilitated through the use of the national electronic health records system in Qatar. Subsequently, employing a stratified random sampling technique, we randomly selected proportionate number of students from each stratum to reach the calculated sample size, ensuring a representative sample of the target population.

Primary data was collected via telephone interviews with the parents or legal guardians of the selected students. Additionally, we retrieved visual acuity measurements from the electronic health records system. Before proceeding with data collection, we obtained verbal consent from each participant, and this consent was duly documented to adhere to ethical guidelines.

### The Data Collection Tool

The questionnaire used in this study comprised two sections. The first section assessed the students’ socio-demographic characteristics and background information, such as their age, nationality, age of their parents, the highest educational level of their mothers and fathers, mother’s employment status, and other general information. The second section of the questionnaire focused on assessing the average screen time for non-academic reasons during both weekdays and weekends. Parents were specifically asked to provide screen time data for two distinct periods: before school closures and during the period of school closures. The questionnaire used was assessed for both face and content validities, as detailed in a previous study (Abed Alah et al., 2023).

To evaluate the impact of school closures on visual acuity, we retrieved pertinent visual acuity measurements from the electronic health records system. This approach enabled us to acquire data encompassing both the period before and after school closures. Utilizing the visual acuity results, we categorized the students into four groups, in accordance with the classification outlined by the World Health Organization (WHO)(World Health Organization (WHO), 2019). The categories were as follows:

1. Normal Visual Acuity: Students with a visual acuity of 6/6.
2. Subnormal Visual Acuity: Students with a visual acuity equal to or worse than 6/9 but better than or equal to 6/12 in the better eye.
3. Mild Visual Impairment: Students with a visual acuity worse than 6/12 but better than or equal to 6/18 in the better eye.
4. Moderate Visual Impairment: Students with a visual acuity worse than 6/18 but better than or equal to 6/60 in the better eye.

By comparing the visual acuity values prior to and subsequent to school closures, we pinpointed students who shifted to more severe visual impairment categories. These individuals were identified as having experienced a decline of their visual acuity, indicating a deterioration in their vision throughout the school closure period.

### Data Analysis

The statistical analysis was conducted using IBM SPSS Statistics for Windows, version 26.0 (IBM Corp., Armonk, NY). Descriptive statistics were used to summarize the data, with percentages reported for categorical variables and mean and standard deviation reported for continuous variables. To compare categorical outcomes between groups, the Chi-square or Fisher’s Exact tests were used as appropriate, while the independent Student’s t-test or Mann-Whitney U test was used to compare continuous and ordinal outcomes as appropriate. We analyzed the change in screen time over the period of school closures using the Paired Student’s t-test. Preliminary univariate logistic regression was used to identify potential predictors of worsening visual acuity. Logistic regression analysis was then conducted, incorporating variables with a univariate association threshold of *p* < 0.25 or with a proven association in previous literature, with adjusted odds ratios (AORs) and 95% confidence intervals (95%CIs) used to report associations between risk factors and outcomes. The Box-Tidwell test was used to assess the assumption of linearity relationship between the independent continuous variable (screen time change), and the logit transformation of the dependent variable (visual acuity change) in the logistic regression. The goodness of fit was assessed using the Hosmer-Lemeshow test, and significance was set at *p* < 0.05.

## Results

### Sociodemographic Characteristics of the Participants

**Fig. 1:**
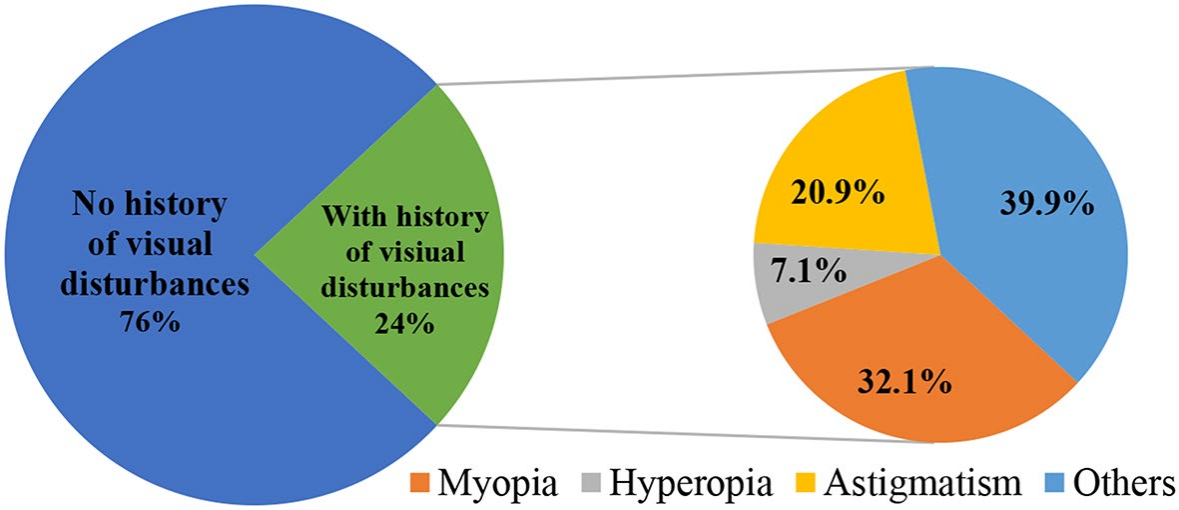
The distribution of visual disturbances of the included students

A total of 1546 interviews were conducted with the parents of the selected group of students, who had an average age of 11 ± 2 years. The sex distribution was almost similar with 49.7% males, and 50.3% females. Within the overall sample, 572 individuals (37%) were local students while the remaining participants were expatriates. According to the data presented in Table 1, around 23.8% (368) of parents indicated that their children had previously been diagnosed with visual disturbances, primarily refractive errors. Notably, over 88% of these parents reported the utilization of eyeglasses or medical contact lenses to manage their children’s conditions. It is worth highlighting that myopia emerged as the most frequently mentioned refractive error, accounting for 32.1% of reported visual disturbances, as illustrated in Fig. 1.

**Table 1:** Sociodemographic characteristics of the included students and background information

Characteristic		No (%)
Student age (M ± SD)		11 ± 2
Student’s age category	8–11 years	845 (54.7)
	12–15 years	701 (45.3)
Gender	Female	777 (50.3)
	Male	769 (49.7)
Nationality	Expatriates	974 (63.0)
	Locals (Qatari)	572 (37.0)
Number of siblings	3 or less	740 (47.9)
	4–6	655 (42.4)
	> 6	151 (9.8)
History of visual disturbances	No	1178 (76.2)
	Yes	368 (23.8)
Wearing eyeglasses	No	1219 (78.8)
	Yes	327 (21.2)
Mother’s age (M ± SD)		40 ± 6
Mother’s age category	< 35	297 (19.6)
	35–44	902 (59.5)
	45–54	303 (20.0)
	55 or more	13 (0.9)
Father’s age (M ± SD)		45 ± 8
Father’s age category	< 35	68 (4.5)
	35–44	688 (45.7)
	45–54	568 (37.8)
	55 or more	180 (12.0)
Mother’s education	No formal education	76 (4.9)
	Primary school level	101 (6.5)
	preparatory school level	126 (8.2)
	Secondary/ high school level	466 (30.1)
	College or higher	777 (50.3)
Father’s education	No formal education	40 (2.6)
	Primary school level	77 (5.0)
	preparatory school level	137 (8.9)
	Secondary/ high school level	414 (26.8)
	College or higher	878 (56.8)
Mother’s employment status	Employed	711 (46.0)
	Not employed	835 (54.0)

*Abbreviations* M, Mean; SD, Standard deviation

### The Impact of School Closures on Screen Time

Excluding the time spent attending online classes, the average screen time for the entire week prior to school closures was reported as 17.4 ± 10.5 h (2.5 ± 1.5 h/day). During the closure period this increased to 28.9 ± 14.1 h/week (4.1 ± 2.0 h/day). In the context of weekdays, the screen time averaged 2.3 ± 1.5 h before closure and escalated to 4.1 ± 2.1 h during the closure. On weekends, the screen time was 3.0 ± 2.1 h before closure and rose to 4.3 ± 2.2 h during the closure as detailed in Table 2. The most commonly used digital device during online classes was Notepad/iPad/Tablet utilized by 42.9% of the total sample.

**Table 2:** Screen time before and during COVID-19-related school closures among children and adolescents in Qatar

Screen time (in hours)		Screen time (in hours)		Change in screen times (in hours)		*P* values*
		M ± SD	Median (IQR)	Change M ± SD	Median (IQR)	
Total weekly screen time (hours/week)	Before school closure	17.4 ± 10.5	14 (10.5–22)	11.5 ± 11.6	10 (0–18)	< 0.001
	During school closure	28.9 ± 14.1	28 (21–37)			
Daily screen time (hours/day)	Before school closure	2.5 ± 1.5	2 (1.5-3)	1.6 ± 1.7	1.5 (0-2.5)	< 0.001
	During school closure	4.1 ± 2.0	4 (3–5)			
On weekdays (hours/day)	Before school closure	2.3 ± 1.5	2 (1–3)	1.8 ± 1.9	1.5 (0–3)	< 0.001
	During school closure	4.1 ± 2.1	4 (3–5)			
On weekends (hours/day)	Before school closure	3.0 ± 2.1	3 (2–4)	1.3 ± 1.7	1 (0–2)	< 0.001
	During school closure	4.3 ± 2.2	4 (3–6)			

*Abbreviations* M, Mean; SD, Standard deviation

*Using Paired Student’s t test

The average screen time over the entire week increased significantly by 11.5 ± 11.6 h (equivalent to a rise of 1.6 ± 1.7 h/day) during school closures in comparison to the period prior to closure excluding the time spent in online classes (*p* < 0.001). The increase was reported in roughly three-quarters of children and adolescents. On weekdays, the average screen time increased by 1.8 ± 1.9 h, while on weekends it increased by 1.3 ± 1.7 h, as shown in Table 2. The mean screen time increased in 71% of participants during a weekday, and in about 56% of participants during a weekend day. The increase in the weekly screen time was statistically significant in the total sample, both sexes and across different age groups. Nonetheless, the mean change (increase) in weekly screen time during the closure was significantly higher among females in comparison to males (13 vs. 10 h, *p* < 0.001) but no significant difference in screen time change was observed across the different age groups as shown in Table 3.

**Table 3:** The changes in screen time over the period of COVID-19-related school closures in the total sample, both sexes, and age groups

Characteristics		Screen time (hours per week)			
		Difference M ± SD	Difference Median (IQR)	*P*-value*	*P*-value†
Total sample		11.5 ± 11.6	**10 (0–18)**	**< 0.001**	---------
Sex	Female	13.0 ± 11.9	**12.5 (5–20)**	**< 0.001**	**< 0.001**
	Male	10 ± 11.2	**7 (0–14)**	**< 0.001**	
Students’ age groups	8–11 years	11.6 ± 11.1	**9 (2–17)**	**< 0.001**	0.746
	12–15 years	11.4 ± 12.3	**10 (0–19)**	**< 0.001**	

*Abbreviations* M, Mean; SD, Standard deviation

* Comparing the screen time before and during schools’ closure using Paired t test

† Comparing the changes of screen time by sex and age group using the independent Student’s t test

### The Impact of School Closures on Visual Acuity

Baseline visual acuity measurements for 253 participants couldn’t be retrieved. Accordingly, these participants were excluded from the analysis of the impact of school closures on visual acuity. The visual acuity results ranged from 6/36 − 6/6 before closure and from 6/60 − 6/6 after closure.

**Fig. 2:**
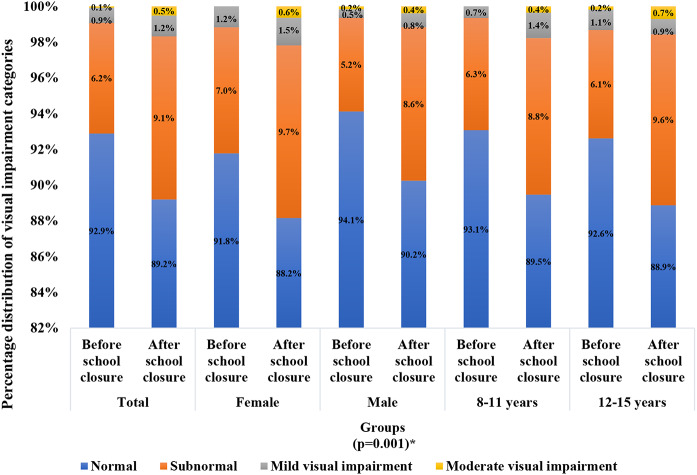
The distribution of visual impairment categories before and after COVID-19-related school closures among governmental students in Qatar

A significant decline of visual acuity was observed based on the visual acuity screening conducted after lifting school closures as compared to before closure in the total sample (*p* = 0.001), both sexes (*p* = 0.025 for females, *p* = 0.021 for males), and within the younger age group (*p* = 0.001). Before the implementation of school closures, 92 out of 1293 (7.1%) exhibited abnormal visual acuity results (defined as a visual acuity worse than 6/6). These cases were further categorized into subnormal (6.2%, denoting visual acuity worse than or equal to 6/9 but better than or equal to 6/12 in the better eye), mild visual impairment (0.9%, indicating visual acuity worse than 6/12 but better than or equal to 6/18 in the better eye), and moderate visual impairment (0.1%, defined as a visual acuity worse than 6/18 but better or equal to 6/60 in the better eye). following the reopening of schools, 167 out of 1546 (10.8%) exhibited abnormal visual acuity (9.1% subnormal, 1.2% mild visual impairment, and 0.5% moderate visual impairment). Using the Wilcoxon matched pair Signed Rank test, we found a significantly higher proportion of children and adolescents categorized in elevated (worsened) visual impairment categories compared to the period before closure (*p* = 0.001) as shown in Fig. 2.

### Determinants and Predictors of Worsening Visual Acuity

Using the chi-square test, we identified significant associations between student sex, history of visual disturbances, and the utilization of eyeglasses or medical contact lenses with worsening visual acuity (Table 4). A greater proportion of local students exhibited worsening visual acuity in comparison to expatriates (11.9% vs. 7.8%, *p* = 0.013). Similarly, students with a history of visual disturbances displayed a higher proportion of worsening visual acuity compared to those without such a history (15.9% vs. 7.3%, *p* < 0.001). Additionally, participants wearing eyeglasses or medical contact lenses reported a higher proportion of worsening visual acuity as opposed to those who didn’t (15% vs. 7.9%, *p* < 0.001).

**Table 4:** The associations between the change in visual acuity over the period of COVID-19-related school closures and the sociodemographic, health-related factors, and screen time changes among children and adolescents in Qatar (*n* = 1293)

Participants’ characteristic		Worsened visual acuity	
		No (%)	*p*-value*
Students’ age categories	Middle childhood (8–11 years)	75 (9.8)	0.508
	Teens and teenagers (12–15 years)	46 (8.7)	
Sex	Female	72 (10.6)	0.114
	Male	49 (8.0)	
Nationality (Qatari, non-Qatari)	Non-Qatari	62 (7.8)	0.013
	Qatari	59 (11.9)	
Number of siblings	3 or less	52 (8.3)	0.363
	4–6	58 (10.7)	
	> 6	11 (8.9)	
Visual disturbances	No	72 (7.3)	< 0.001
	Yes	49 (15.9)	
Wearing eyeglasses or medical contact lenses	No	80 (7.9)	< 0.001
	Yes	41 (15)	
Mothers’ age categories	< 35	28 (11.2)	0.604
	35–44	65 (8.6)	
	45–54	26 (10.5)	
	55 or more	1 (10%)	
Fathers’ age categories	< 35	8 (12.5)	0.623
	35–44	57 (10)	
	45–54	40 (8.4)	
	55 or more	12 (8.1)	
Mother’s education	No formal education	2 (3.6)	0.445
	Primary school level	9 (10.8)	
	preparatory school level	10 (9.8)	
	Secondary/ high school level	43 (10.8)	
	College or higher	57 (8.7)	
Father’s education	No formal education	1 (3.2)	0.703
	Primary school level	6 (9.5)	
	preparatory school level	12 (10.9)	
	Secondary/ high school level	30 (8.5)	
	College or higher	72 (9.8)	
Mother’s employment	Employed	58 (9.7)	0.696
	Not employed	63 (9.1)	
Change in screen time (M ± SD)		11.9 ± 11.3	0.817

*Abbreviations* M, Mean; SD, Standard deviation

* Using the Chi-square or Fisher Exact tests to assess the associations with categorical variables and the independent

Student’s t-test to assess the association with the change in screen time

We carried out a logistic regression model to assess the predictors of worsening visual acuity. The model demonstrated a good fit and indicated that local students with history of visual disturbances were 1.7 times (AOR: 1.73, 95%CI 1.18–2.54, *p* = 0.005), and 2.5 times (AOR: 2.52, 95%CI 1.69–3.76, *p* < 0.001) more likely, respectively to experience worsening of visual acuity over the period of school closures compared to expatriate students without a history of visual disturbances (Table 5).

**Table 5:** The sociodemographic and health-related predictors of the change in visual acuity over the period of COVID-19-related school closures among children and adolescents in Qatar (*n* = 1293)

Characteristics		Worsened visual acuity				
		Unadjusted OR (95%CI)	*P*-value	F test *P*-value	AOR (95%CI)	*P*-value
Students’ age categories	Middle childhood (8–11 years)	1.14 (0.78–1.67)	0.508	0.508	1.31 (0.88–1.94)	0.183
	Teens and teenagers (12–15 years)	1 [Reference]			1 [Reference]	
Sex	Female	1.36 (0.93–1.99)	0.115	0.115	1.29 (0.88–1.91)	0.197
	Male	1 [Reference]			1 [Reference]	
Nationality (Qatari, non-Qatari)	Non-Qatari	0.63 (0.43–0.91)	0.014	0.014	0.58 (0.39–0.85)	0.005
	Qatari	1 [Reference]			1 [Reference]	
Number of siblings	3 or less	1 [Reference]		0.365	-------	-----
	4–6	1.33 (0.89–1.96)	0.161		-------	-----
	> 6	1.08 (0.55–2.13)	0.832		-------	-----
Visual disturbances	No	1 [Reference]		< 0.001	1 [Reference]	
	Yes	2.39 (1.62–3.52)	< 0.001		2.52 (1.69–3.76)	< 0.001
Wearing eyeglasses or medical contact lenses	No	1 [Reference]		< 0.001	-------	**-----**
	Yes	2.07 (1.38–3.09)	< 0.00		-------	**-----**
Mothers’ age categories	< 35	1 [Reference]		0.608	-------	**-----**
	35–44	0.75 (0.47–1.20)	0.224		-------	**-----**
	45–54	0.94 (0.53–1.65)	0.821		-------	**-----**
	55 or more	0.89 (0.11–7.25)	0.909		-------	**-----**
Fathers’ age categories	< 35	1 [Reference]		0.626	-------	**-----**
	35–44	0.78 (0.35–1.01)	0.526		-------	**-----**
	45–54	0.64 (0.29–1.45)	0.286		-------	**-----**
	55 or more	0.62 (0.24–1.59)	0.319		-------	**-----**
Mother’s education	No formal education	0.40 (0.09–1.67)	0.207	0.475	-------	**-----**
	Primary school level	1.28 (0.61–2.69)	0.518		-------	**-----**
	Preparatory school level	1.14 (0.56–2.32)	0.712		-------	**-----**
	Secondary/ high school level	1.28 (0.84–1.94)	0.251		-------	**-----**
	College or higher	1 [Reference]			-------	**-----**
Father’s education	No formal education	0.31 (0.04–2.28)	0.249	0.730	-------	**-----**
	Primary school level	0.97 (0.40–2.33)	0.944		-------	**-----**
	Preparatory school level	1.13 (0.59–2.15)	0.716		-------	**-----**
	Secondary/ high school level	0.85 (0.55–1.33)	0.484		-------	**-----**
	College or higher	1 [Reference]			-------	**-----**
Mother’s employment	Employed	1.08 (0.74–1.57)	0.696	0.696	-------	**-----**
	Not employed	1 [Reference]			-------	
Screen time change		1.00 (0.97–1.02)	0.817		1 (0.98–1.02)	0.975

*Abbreviations* OR, Odds Ratios AOR, Adjusted Odds Ratio; CI, Confidence interval

## Discussion

The global onset of COVID-19 pandemic compelled countries across the globe to undertake essential measures to curb its transmission, including the closure of educational institutions, most notably schools. Consequently, traditional face-to-face teaching methods were substituted with virtual online classes, compelling students to spend extended periods in front of screens. Besides attending online classes, children and adolescents increasingly engaged with digital devices for leisure pursuits and social interactions, including social media usage (Pandya & Lodha, n.d.). The prolonged and excessive use of screens among children and adolescents yields significant repercussions for their physical and mental well-being. Firstly, excessive screen time has been linked to sleep disturbances and psychological issues. Extended exposure to screens, particularly before bedtime, can disrupt sleep patterns and result in sleep-related problems (Singh & Balhara, 2021). It’s noteworthy that most existing studies don’t clearly specify whether their evaluation of screen time during the pandemic encompassed the duration spent in online classes thus potentially impacting the precision of comparing our findings with available literature. In our study, we observed a mean increase of 1.6 ± 1.7 h/day of average screen time which is comparatively lower than what was reported in studies from Spain (2.9 h), and Tunisia (2.8 h) (Abid et al., 2021; López-Bueno et al., 2020). This discrepancy could potentially be elucidated by the fact that those studies evaluated screen time in a broader sense and didn’t explicitly indicate whether they accounted for time spent in online classes as mentioned earlier. In a recent metanalysis of 89 studies, only three studies conducted in Singapore, China, and the United States of America specifically detailed and reported the change in leisure non-academic screen time. These studies revealed an increase ranging from 0.5 to 1.0 h/day which is comparatively less than the change observed in our study (Trott et al., 2022).

In our study, we observed a significant decline in visual acuity among students over the period of school closures, a finding consistent with results reported by other investigations (Arulappan et al., 2022; Noi et al., 2022).Refractive errors emerged as the prevailing cause of visual impairment, accounting for approximately 60% of reported visual disturbances, with myopia standing as the predominant refractive error. Existing evidence from a recent systematic review and meta-analysis corroborates the association between COVID-19-related lockdown measures and an elevated risk of myopia progression in children. This linkage is primarily attributed to excessive screen time and reduced outdoor activities during the pandemic (Yang et al., 2022). Another meta-analysis revealed that spending more time outdoors was associated with a 2% reduction in the odds of developing myopia for each extra hour spent outdoors per week, even after adjusting for other factors, among children and adolescents (Sherwin et al., 2012). Additionally, a study investigating risk factors associated with myopia in school-aged children identified a significant association between screen time exceeding 3 h per day and reduced sun exposure during summertime (Harrington et al., 2019). The transition from outdoor activities to indoor screen-based activities, such as watching television, playing video games, and engaging in social media, triggered by school closures inevitably contributed to the escalation of screen time among children and adolescents. Researchers worldwide are advocating for the implementation school-based initiatives aimed at preventing and mitigating the progression of myopia by encouraging children to spend more time engaging in outdoor activities (Sherwin et al., 2012; Wu et al., 2018). However, implementing such programs in Qatar could present challenges due to the country’s hot and humid climate, particularly during summer season. The extreme weather conditions might hinder the feasibility of outdoor activities and thereby influence the applicability of this recommendation in the Qatari context.

The interruption of healthcare services and the transition to teleconsultation during the COVID-19 pandemic may have contributed to a significant decline in visual acuity among students particularly those with previous history of visual disturbances who are in need of timely follow ups. With limited access to in-person healthcare, students might have faced delays in the detection and management of abnormal visual acuity, potentially exacerbating visual problems during the school closure period. Notably, the visual acuity screening at schools was also disrupted during the COVID-19-related school closures in Qatar. This further hindered the timely identification and intervention for students with vision issues, potentially leading to the observed worsening of visual acuity.

Additionally, our study revealed that local students of Qatari nationality demonstrated a higher likelihood of experiencing deteriorated visual acuity when compared to their expatriate peers. This difference may be explained by the significantly higher screen time observed among local students both prior to and during the closure. Local students reported dedicating more time to screen based activities (19.3 vs. 16.3 h/week before the closure and 30.7 vs. 27.9 h/week during the closure, *p* < 0.001) when compared to their expatriate counterparts. Being exposed to higher levels of screen time for a longer period that started before the closure might have contributed to a higher decline in visual acuity. Moreover, the discrepancy might reflect that certain underlying genetic or biological differences can contribute to the progression of visual disturbances more rapidly in some populations.

This discrepancy in screen time between local and expatriate students could be attributed to the overall better socioeconomic status of Qatari students in general. Their improved financial standing may render them more likely to possess smartphones, laptops, and video game consoles, which, in turn, contributes to increased screen time within this group.

Although this study did not find a significant association between the amount of change (increase) in screen time and the worsening of visual acuity, it is not conclusive evidence that screen time has no impact on visual health. The duration of school closures and the timeframe of data collection in this study might not have captured long-term effects of screen time change on visual acuity. Moreover, while excessive screen time has been associated with various visual issues, such as digital eye strain and screen-induced foveal dysfunction, the specific influence on visual acuity can be influenced by multiple factors, including individual variations in visual health and the type of visual tasks performed during screen time (Rosenfield, 2011).

During school closures, it is crucial to take measures to protect children’s vision and reduce their screen time. Encouraging regular breaks during screen time is a vital step towards achieving this goal. It is recommended to schedule breaks after every 20–30 min of screen time, during which children should stand up, stretch, and look away from the screen for a few minutes. Parents should act as role models for their children by reducing their own screen time and engaging in shared activities with them. They should also be encouraged to use screen control applications to monitor and limit their children’s screen time. In addition, promoting outdoor activities effectively reduces screen time and provides opportunities for physical exercise and exposure to natural light, which is essential for good vision. It is also important to establish healthy screen time habits, such as maintaining a safe distance from the screen, adjusting screen brightness, and avoiding the use of screens before bedtime. Providing alternative activities such as reading books, playing board games, or engaging in creative activities like drawing or painting can also help reduce screen time. Furthermore, it is recommended to schedule regular eye checkups to ensure that any vision problems are detected early and treated appropriately. By implementing these measures, parents and educators can protect children’s vision and promote healthy screen time habits during school closures.

This study had several strengths, such as a suitable sampling technique and adequate sample size. The study’s visual acuity measurements were sourced from a reputable and credible data source. However, the retrospective approach employed to gather screen time-related data from parents might have introduced some recall bias. Furthermore, the potential influence of social desirability bias on parents’ responses cannot be overlooked. Parents may have underreported their children’s actual screen time due to societal expectations or concerns about excessive screen usage, leading to an underestimation of the true extent of screen time among the participants.

## Conclusion

The results of this study indicate a notable reduction in visual acuity among children and adolescents aged 8 to 15 during the period of school closures. Concurrently, there was a significant increase in screen time among this population. These findings highlight the potential negative impact of school closures on screen time habits and visual acuity, especially during prolonged periods of remote learning. The study highlights the importance of implementing strategies to reduce screen time and protect visual health, particularly in the context of extended remote learning scenarios. Addressing excessive screen time and promoting healthy technology use should be integral components of educational and public health initiatives. Future research is warranted to gain a deeper understanding of the long-term effects of screen time on visual health and to inform evidence-based recommendations for promoting optimal screen usage habits among children and adolescents.

## Data Availability

Data will be available upon reasonable request from the corresponding author.
